# Artesunate Alleviates Hyperoxia-Induced Lung Injury in Neonatal Mice by Inhibiting NLRP3 Inflammasome Activation

**DOI:** 10.1155/2023/7603943

**Published:** 2023-02-04

**Authors:** Bin Xie, Shouye Li, Wuxia Bai, Zheming Li, Feifeng Lou

**Affiliations:** ^1^Department of Pharmacy, Shaoxing Integrated Traditional Chinese and Western Medicine Hospital, Shaoxing, China; ^2^College of Pharmacy, Hangzhou Medical College, Hangzhou, China; ^3^Department of Neonatology, Zhuji People's Hospital of Zhejiang Province, Zhuji, China

## Abstract

Bronchopulmonary dysplasia (BPD) is a chronic respiratory disease in preterm infants that may cause persistent lung injury. Artesunate exhibits excellent anti-inflammatory in lung injury caused by various factors. This study aimed to investigate the effect of the artesunate on hyperoxia-induced lung injury in neonatal mice and its mechanism. A BPD model of hyperoxic lung injury in neonatal mice was established after hyperoxia (75% oxygen) exposure for 14 days, and part of the mice received intraperitoneal injections of the artesunate. H&E staining was used to observe the pathology of lung tissue, and the degree of oxidative stress in the lung tissue was determined by commercial kits. The levels of inflammatory cytokines in the serum and lung tissues of neonatal mice were detected by an enzyme-linked immunosorbent assay. Immunohistochemical experiments were performed to further evaluate the expression of IL-1*β*. The real-time quantitative polymerase chain reaction was used to determine the mRNA level of the NLRP3 inflammasome. The western blot assay was used to measure the levels of NLRP3 inflammasome and NF-*κ*B pathway-related proteins. Artesunate ameliorated weight loss and lung tissue injury in neonatal mice induced by hyperoxia. The level of malondialdehyde was decreased, while the activity of superoxide dismutase and the level of glutathione increased after artesunate treatment. Artesunate reduced the level of inflammation cytokines TNF-*α*, IL-6, and IL-1*β* in the serum and lung. Moreover, artesunate inhibited the mRNA expression and protein levels of NLRP3, ASC, and caspase-1, as well as the phosphorylation of the NF-*κ*B and I*κ*B*α*. Our findings suggest that artesunate treatment can attenuate hyperoxia-induced lung injury in BPD neonatal mice by inhibiting the activation of NLRP3 inflammasome and the phosphorylation of the NF-*κ*B pathway.

## 1. Introduction

Recent studies have shown that the incidence of bronchopulmonary dysplasia (BPD) is increasing and can reach 30% among extremely premature infants born before 28 weeks of gestational age [1, 2]. Children with BPD often have a poor prognosis and suffer from repeated respiratory infections and even a gradual decline in lung function with age [3]. BPD is related to a variety of factors, including adverse prenatal stimuli such as placental hypoxia caused by preeclampsia, the risk of premature birth, and postpartum adverse stimuli such as mechanical ventilation, hyperoxia, and lung injury caused by inflammation [4]. Hyperoxia exposure is the main risk factor for premature infants suffering from BPD, and due to their incomplete pulmonary function and antioxidant stress system and lack of lung surfactant, they are more susceptible to oxidative stress and inflammatory damage caused by hyperoxia environment after birth [3]. NLRP3 inflammasome is closely related to the development of BPD. Studies have shown that mice with a deletion of the NLRP3 gene do not have lung inflammation and damage during hyperoxia exposure [5]. The main components of NLRP3 inflammasome include the sensor molecule NLRP3, apoptosis-associated speck-like protein containing a CARD (ASC), and effector protease caspase-1 [6]. The combination of NLRP3 and ASC activates caspase-1 to trigger the activation and release of the IL-1 family of inflammatory cytokines, among which IL-1*β* was confirmed to be overexpressed in the alveolar epithelial cells of BPD neonatal mice [7]. With the development of neonatal medicine and perinatal care, the survival rate of preterm infants has improved continuously, but the high incidence of BPD has persisted [8]. At present, a variety of treatments for BPD have been proposed, including ventilation strategies [9], peroxisome proliferator-activated receptor (PPAR) *γ* agonists [10], stem cell therapy [11], and so on. However, these therapies are still in preclinical or clinical trials, in which clinical efficacy and exact safety still need to be evaluated. Some drugs that have been shown to be effective for lung injury may be used in the treatment of BPD.

Artesunate is a water-soluble hemisuccinate derivative of the famous antimalarial drug artemisinin, which has been proven to have a variety of biological functions, including inhibiting tumor growth [12, 13], immunomodulatory function [14], and anti-inflammatory and antioxidative stress effects in a variety of respiratory diseases [15]. Liu et al. [16] reported that artesunate pretreatment reduces the activation of NLRP3 inflammasomes induced by ROS to attenuate acute lung injury mediated by renal ischemia-reperfusion. In addition, artesunate activated the NF-*κ*B signaling pathway in a dose-dependent manner [17], reduces the production of inflammatory cytokines TNF-*α*, IL-1*β*, and IL-6, and enhanced the activities of the antioxidant enzymes myeloperoxidase and heme oxygenase-1, thereby alleviating LPS-induced acute lung injury [18]. Artesunate has exhibited excellent anti-inflammatory and antioxidative stress effects in lung injury caused by various reasons, but its role in hyperoxia-induced lung injury and the underlying mechanism has not been reported. In the present study, we established a BPD neonatal mouse model to investigate the effect of artesunate on hyperoxia-induced lung inflammation and lung injury. The effects of artesunate on oxidative stress and inflammatory response in lung tissues and the underlying mechanisms were further evaluated and emphasized on the role of NPRL3 inflammasome.

## 2. Materials and Methods

### 2.1. Main Reagents

4% paraformaldehyde, hematoxylin and eosin (H&E) dye solution set, citrate (pH 6.0) antigen retrieval solution, immunohistochemical kit 3,3-N-diaminobenzidine tertrahydrochloride (DAB) chromogenic agent, phosphate-buffered saline (PBS) buffer, and bovine serum albumin (BSA, G5001) werepurchased from Servicebio (Wuhan, China). Ethanol absolute, xylene, neutral balsam, and hydrogen peroxide 3% aqueous solution were purchased from Sinopharm Chemical Reagent Co., Ltd. (Shanghai, China). Antibody IL-1*β* (DF6251), p-NF-kB p65 (AF2006), NF-kB p65 (AF5006), p-IkB*α* (AF2002), IkB*α* (AF5002), NLRP3 (DF7438), ASC (DF6304), caspase-1 (AF5418), caspase-3 (AF6311), GAPDH (AF7021), and horseradish peroxidase (HRP)-labeled rabbit anti-goat IgG (S0010) were purchased from Affinity Biosciences (OH, USA). An enzyme-linked immunosorbent assay (ELISA) kit was purchased from Jiangsu Meimian Industrial Co., Ltd. (Jiangsu, China). Malondialdehyde (MDA) assay kit, superoxide dismutase (SOD) assay kit, and reduced glutathione (GSH) assay kit were purchased from Nanjing Jiancheng Bioengineering Institute (Nanjing, China). The bicinchoninic acid (BCA) protein assay kit was purchased from Solarbio (Beijing, China). Radioimmunoprecipitation assay (RIPA), lysis buffer, and phenylmethanesulfonyl fluoride (PMSF) were purchased from Beyotime Biotechnology (Shanghai, China). The enhanced chemiluminescence (ECL) kit was purchased from Clinx (Shanghai, China). A reverse transcription kit was purchased from Xilong Scientific (Shantou, China).

### 2.2. Animals and Study Design

Adult (6–8 weeks old) C57BL/6 mice were purchased from the Animal Experiment Center of Hangzhou Eyong Biotechnological Co., Ltd. (No. SYXK (Zhe) 2020–0024). All animal experiments complied with the Recommended Guideline for the Care and Use of Laboratory Animal Research by the Chinese Council on Animals, approved by the Animal Ethics Committee of the Hangzhou Eyong Biomedical Co., Ltd. Adult mice were hybridized to obtain neonatal mice for subsequent studies. At the expiration of pregnancy (days 21 to 22), the dams naturally gave birth, and 24 male neonatal mice were randomly divided into four groups with six neonatal mice in each group: the normoxia (NO) group; normoxia + artesunate (NA) group; hyperoxia (HO) group; hyperoxia + artesunate (HA) group. Hyperoxia-induced lung injury in neonatal mice models was established according to the methods described in a previous study [19]. With continuous monitoring of indoor O_2_ concentration using an oxygen analyzer in a plexiglass chamber, the mice in the NO and NA groups were exposed to 21% O_2_, while the mice in the HO and HA groups were exposed to 75% O_2_. The neonatal mice in the NA and HA groups were given an intraperitoneal injection of artesunate (15 mg/kg) every other day as described previously [16], while mice in the NO and HO groups received intraperitoneal injections of saline in equal amounts. All treatments lasted from postnatal day 0 (P0) to P14. To prevent oxygen toxicity, the oxygen environment of the lactating mice was rotated every 24 hours, i.e., mice in the HO group and NO group were rotated, while in the NA group and HA group were rotated. All the neonatal mice survived throughout the experiment. Neonatal mice in each group were weighed at P7 and P14, respectively, and were euthanized by intraperitoneal injection of 1% pentobarbital (50 mg/kg) at P14.

### 2.3. Evaluation of Lung Oxidative Stress

After washing the lung tissues of mice with normal saline at 4°C and absorbing water with filter paper, 100 mg of lung tissues were cut to make 10% homogenate. The levels of oxidative stress in mice lung tissue were detected using the commercial MDA kit, SOD kit, and GSH kit according to the manufacturer's instructions.

### 2.4. Evaluation of Lung Inflammation

Lung tissue was fixed with 4% paraformaldehyde for 48 h followed by embedded with paraffin and sliced into 4 *μ*m sections, which were performed for H&E staining and immunohistochemistry. After dewaxing, sections were stained with H&E and observed and analyzed under a microscope. The inflammation of histopathological results was scored according to the infiltration area of the inflammatory cells, and the swelling and shedding of epithelial cells [20–22]. The grading system was as follows: 0 = no obvious inflammatory cells infiltration and epithelial cells swelling and shedding; 1 = a few inflammatory cells infiltration and no epithelial cells swelling and shedding; 2 = obvious inflammatory cells infiltration and a few epithelial cells swelling and shedding; 3 = obvious inflammatory cells infiltration and a large number of epithelial cells swelling and shedding; 4 = a large number of inflammatory cells infiltration and a large number of epithelial cells swelling and shedding. The scoring process was conducted by three colleagues who were not aware of the experiment, and the average scores were used to represent the inflammation scoring of lung tissue in mice.

### 2.5. Measurement of Inflammatory Factors

The levels of inflammatory factors TNF-*α*, IL-6, and IL-1*β* were measured *via* ELISA. Blood was collected from the right ventricle immediately after the neonatal mice were euthanized. After centrifugation at 3500 rpm for 15 min, the supernatant was stored at −80°C. The lung tissue of the mice was homogenized after adding PBS, centrifuged at 2000 rpm for 20 min, and collecting the supernatant for the following assay. ELISA kits were used to measure the levels of TNF-*α*, IL-6, and IL-1*β* in the serum and lung tissue supernatant according to the manufacturer's instructions. Within 15 minutes after the termination of coloration, the absorbance (OD) value of each specimen hole at 450 nm was determined by using an enzyme-labeled instrument.

### 2.6. Assessment of IL-1*β* Expression Level

Immunohistochemistry was used to assess the IL-1*β* expression level. The degreased and rehydrated lung tissue sections were placed in a repair box within a citric acid antigen repair buffer (pH 6.0) for antigen repair in a microwave oven. The antigen was repaired with medium fire for 8 min to boiling, and the medium-low fire lasted for 7 min after 8 min of the ceasefire. Excessive evaporation of the buffer should be prevented. After natural cooling, sections were incubated in a 3% hydrogen peroxide solution at room temperature for 25 min to block endogenous peroxidase. 3% BSA was added dropwise to the sections for serum blocking at room temperature for 30 min. After the sealing solution was removed by absorbent paper, the sections were incubated overnight with IL-1*β* primary antibody in a wet box at 4°C. Rabbit anti-goat secondary antibody labeled with HRP was added and incubated at room temperature for 50 min. A DAB chromogenic agent was added to the histochemical circle and incubated in the dark for 25 min. The nucleus was restained with hematoxylin for 3 min, and the sections were sealed after dehydration. Image-Pro Plus (Media Cybernetics, USA) software was used for quantitative analysis after microscopic examination.

### 2.7. Assessment of NLRP3 Inflammasome-Related mRNA Levels

NLRP3 inflammasome-related mRNA levels were assessed via real-time quantitative polymerase chain reaction (RT-qPCR). 1000 *μ*l Trizol reagent was added to the lysate total RNA, and prepared the reaction system according to the instructions and using PCR apparatus (Eppendorf, Hamburg, and German) to perform reverse transcription. The reaction conditions were as follows: synthesis of cDNA at 42°C for 15 min; amplification at 85°C for 5 min. The reaction procedure was carried out according to the following conditions on the RT-qPCR instrument (Bio-Rad Laboratories, Hercules, USA): predenaturation at 95°C for 10 min; denaturation at 95°C for 15 s; extension at 60°C for 60 s; 40 cycles. The relative quantitative results were evaluated using the 2^−ΔΔCt^ method and normalized with GAPDH. The primer sequence was as follows: mouse NLRP3 forward, ATCTTTGCTGCGATCAACAG; Mouse NLRP3 reverse, TGATGTACACGTGTCATTCCA; mouse ASC positive, GATCCTCTCGACCTGCTTCG; mouse ASC reversed, CAACACCAAAAGGGCTGCTC; mouse caspase-1 positive, CGTACACGTCTTGCCCTCAT; mouse caspase-1 reversed, GGTCACCCTATCAGTGG; mouse GAPDH forward, ACTCTTCCACCTTCGATGC; mouse GAPDH reverse, CCGTATTCATTGTCATACCAGG.

### 2.8. Assessment of NLRP3 Inflammasome and NF-*κ*B Pathway-Related Proteins

The western blot assay was used to detect the expression levels of the NLRP3 inflammasome and NF-*κ*B pathway-related proteins. Briefly, precooled RIPA cracking solution containing PMSF was added to the clipped lung tissue and homogenized, followed by static cracking on ice for 20 min and centrifugation at 12000 rpm at 4°C for 5 min. The protein content of the supernatant was determined by the BCA protein assay kit. The protein samples were separated by sodium dodecyl sulphate-polyacrylamide gel electrophoresis (SDS-PAGE) and transferred to PVDF membranes, which were sealed with 0.2% Tween-20 Tris-buffered saline (TBST) containing 5% skim milk for 2 h. The samples were incubated with the following primary antibodies at 4°C overnight: NF-*κ*B p65 (1 : 1000), NF-*κ*B P65 (1 : 1000), p-I*κ*B*α* (1 : 1000), IkB*α* (1 : 1000), NLRP3 (1 : 1000), ASC (1 : 1000), caspase-1 (1 : 1000), caspase-3 (1 : 1000), and GAPDH (1 : 3000). After washed with TBST for 3 times, the protein samples were added with HRP-labeled rabbit anti-goat IgG secondary antibody (1 : 5000) and incubated at room temperature for 2 h. The immune response bands interacted with the ECL kit and were visualized on a gel imaging system. The bands were analyzed using the ImageJ software. Using GAPDH as an internal reference, the ratio of each protein to GADPH was calculated to reflect the expression level of the target protein.

### 2.9. Statistical Analysis

SPSS 20.0 statistical software (SPSS Inc., Chicago, USA) was used for data analysis. All data in accordance with the normal distribution were expressed as mean ± standard deviation, and the measurement data among the multiple groups were tested by one-way analysis of variance (ANOVA) and the Student-Newman-Keuls (SNK) test. The Kruskal–Wallis H test was used for those with uneven variance. The results were shown by GraphPad Prism 8.0 (GraphPad Software, San Diego, USA). Values of *P* < 0.05 were considered statistically significant.

## 3. Results

### 3.1. Artesunate Reduced Hyperoxia-Induced Oxidative Stress in BPD Neonatal Mice Lung

**Figure 1:**
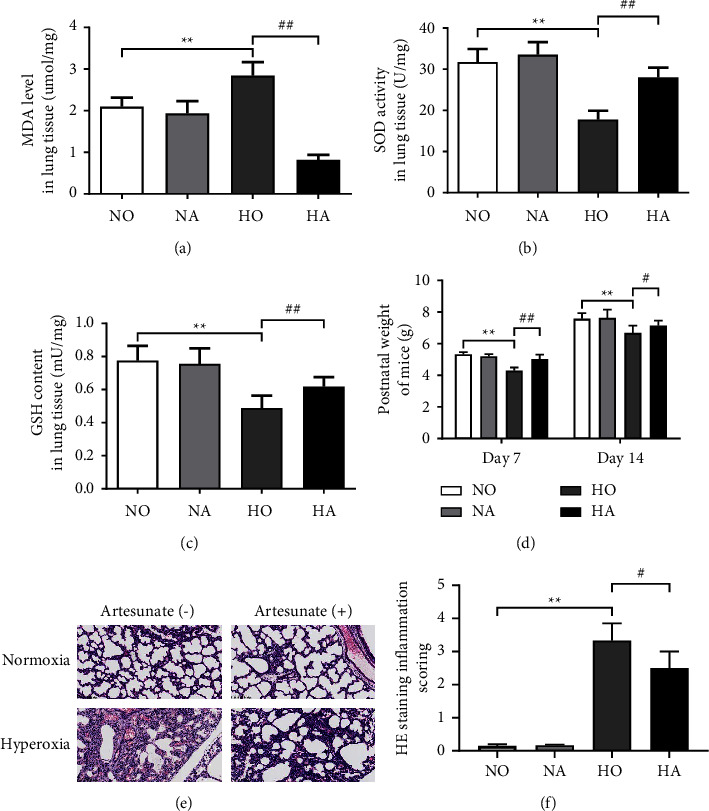
(a–c) The MDA level, SOD activity, and GSH level in the lung tissue of neonatal mice. (d) The body wight of neonatal mice at postnatal day 7 and day 14. (e) H&E staining sections of neonatal mice lung tissue after different treatments (×200). (f) Inflammation score of H&E staining sections. (NO, normoxia group; NA, normoxia + artesunate group; HO, hyperoxia group; HA, hyperoxia + artesunate group. *P* value was calculated by one-way ANOVA and SNK test. ^*∗∗*^*P* < 0.01 versus NO group; ^#^*P* < 0.05, ^##^*P* < 0.01 versus HO group).

MDA level, SOD activity, and GSH activity were measured to evaluate the degree of oxidative stress in the lung tissue of neonatal mice under hyperoxia. Compared with the NO group, the increase of MDA level in the HO group was significant (*P* < 0.01) (Figure 1(a)), while SOD activity and GSH level significantly decreased (*P* < 0.01) (Figures 1(b) and 1(c)), indicating that the lung tissue of neonatal mice showed peroxidation. After artesunate treatment, the MDA level in the HA group was lower than that in the HO group (Figure 1(a)), while SOD activity and GSH levels were increased significantly (*P* < 0.01) (Figures 1(b) and 1(c)), indicating that artesunate could reduce hyperoxia-induced oxidative stress in the lungs of neonatal mice.

### 3.2. Artesunate Ameliorated Hyperoxia-Induced Weight Loss in BPD Neonatal Mice

As shown in Figure 1(d), the body weight of neonatal mice in the NA group did not change significantly compared with that in the NO group, indicating that artesunate may have no obvious acute toxicity to mice. After 7 days of 75% oxygen exposure, the weight of mice in the HO group was significantly reduced (*P* < 0.01), while the weight of neonatal mice in the HA group increased at P7 (*P* < 0.01). Similar weight changes were observed in P14, indicating that HA could ameliorate hyperoxia-induced weight loss in neonatal mice.

### 3.3. Artesunate Attenuated Hyperoxia-Induced Lung Inflammation in BPD Neonatal Mice

The results of H&E staining (Figure 1(e)) showed that the lung tissue structure of mice in the NO group was intact without obvious abnormalities, while that in the NA group was intact and the alveolar structure was clear as well. Interstitial edema and thickened alveolar walls with neutrophils and macrophages infiltrated were observed in the HO group. After being treated with AS, the alveolar structure in the HA group was clear with the alleviation of inflammatory cell infiltration and interstitial edema. As shown in Figure 1(f), the inflammation score in the HO group was significantly higher than that in the NO group (*P* < 0.01), while the inflammation score in the HA group was lower than that in the HO group (*P* < 0.05).

**Figure 2:**
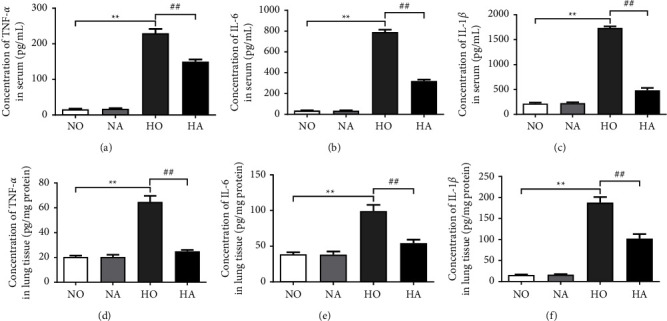
(a–c) Concentrations of TNF-*α*, IL-6, and IL-1*β* in the serum of neonatal mice were detected *via* ELISA. (d–f) Concentrations of TNF-*α*, IL-6, and IL-1*β* in the lung tissue of neonatal mice were detected *via* ELISA. (NO, normoxia group; NA, normoxia + artesunate group; HO, hyperoxia group; HA, hyperoxia + artesunate group. *P* value was calculated by one-way ANOVA and SNK test. ^*∗∗*^*P* < 0.01 versus NO group; ^##^*P* < 0.01 versus HO group).

Moreover, the levels of inflammatory factors in serum and lung tissues were detected *via* ELISA (Figure 2). The levels of TNF-*α*, IL-6, and IL-1*β* in the serum and lung tissue of mice in the HO group were significantly increased (*P* < 0.01), while those in the HA group were significantly lower than those in the HO group (*P* < 0.01). These experimental results suggested that artesunate could reduce hyperoxia-induced lung inflammation in neonatal mice.

### 3.4. Artesunate Decreased Hyperoxia-Induced IL-1*β* Expression in Lung Tissue of BPD Neonatal Mice

**Figure 3:**
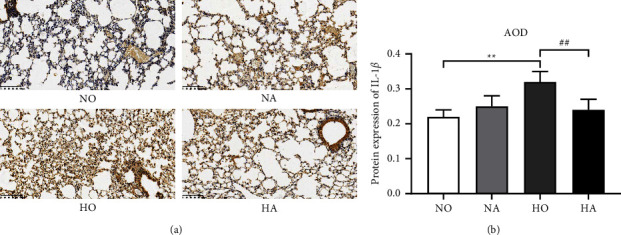
IL-1*β* level was determined by immunohistochemistry. (a) Light microscope results of IL-1*β* level of immunohistochemistry (×200). (b) Area of integral optical density ratio (AOD) of IL-1*β* expression. (NO, normoxia group; NA, normoxia + artesunate group; HO, hyperoxia group; HA, hyperoxia + artesunate group. *P* value was calculated by one-way ANOVA and SNK test. ^*∗∗*^*P* < 0.01 versus NO group; ^##^*P* < 0.01 versus HO group).

Since it was found by ELISA that artesunate could reduce the content of IL-1*β* in the lung tissue of BPD neonatal mice, the expression of IL-1*β* was further detected by immunohistochemistry, and the area of the integral optical density ratio (AOD) was measured (Figure 3). The AOD in the HO group was significantly higher than that in the NO group, while the AOD in the HA group was lower than that in the HO group (*P* < 0.01), indicating that artesunate decreased IL-1*β* expression in the lung tissue of hyperoxia-induced neonatal BPD mice.

### 3.5. Artesunate Reduced Hyperoxia-Induced Activation and Expression of NLRP3 Inflammasome

**Figure 4:**
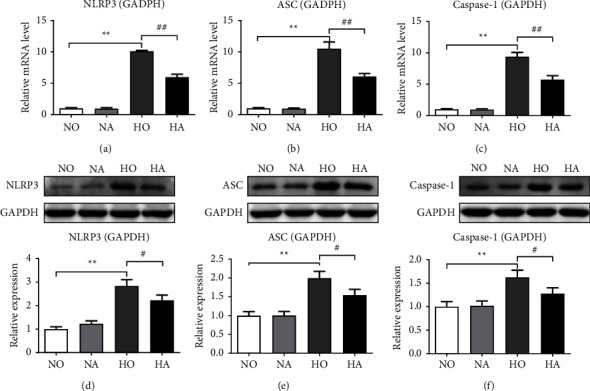
mRNA and protein expression levels of NLRP3 inflammasome. (a–c) Relative mRNA levels of NLRP3, ASC, and caspase-1 were measured *via* RT-qPCR. (d–f) Relative expression levels of NLRP3, ASC, and caspase-1 were measured *via* western blot assay. (NO, normoxia group; NA, normoxia + artesunate group; HO, hyperoxia group; HA, hyperoxia + artesunate group. *P* value was calculated by one-way ANOVA and SNK test. ^*∗∗*^*P* < 0.01 versus NO group; ^#^*P* < 0.05, ^##^*P* < 0.01 versus HO group).

The mRNA levels of NLRP3, ASC, and caspase-1 were measured *via* RT-qPCR. As shown in Figures 4(a)–4(c), results demonstrated that the relative mRNA levels of the main components of NLRP3 inflammasome in the HO group increased compared with those in the NO group (*P* < 0.01). After treatment with AS, the relative mRNA levels of NLRP3, ASC, and caspase-1 in the HA group were lower than those in the HO group, indicating that artesunate could reduce hyperoxia-induced activation of the NLRP3 inflammasome.

The western blot assay was used to evaluate the expression of NLRP3 inflammasome-related proteins. As shown in Figures 4(d)–4(f), the relative expression of NLRP3, ASC, and caspase-1 in the lung tissue of mice was significantly increased after hyperoxia exposure for 14 days (*P* < 0.01). Compared with the HO group, the relative expression of NLRP3 inflammasome in the HA group was significantly decreased (*P* < 0.05), indicating that artesunate could reduce hyperoxia-induced expression of NLRP3 inflammasome.

### 3.6. Artesunate Inhibited Hyperoxia-Induced Phosphorylation of the NF-*κ*B Pathway

**Figure 5:**
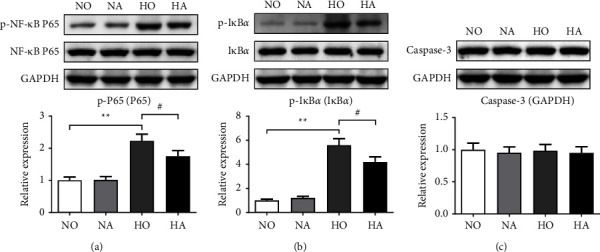
Relative expression levels of NF-*κ*B pathway-related proteins were measured *via* western blot assay. (a) Relative expression levels of p-NF-*κ*B p65. (b) Relative expression levels of p-I*κ*B*α*. (c) Relative expression levels of caspase-3. (NO, normoxia group; NA, normoxia + artesunate group; HO, hyperoxia group; HA, hyperoxia + artesunate group. *P* value was calculated by one-way ANOVA and SNK test. ^*∗∗*^*P* < 0.01 versus NO group; ^#^*P* < 0.05 versus HO group).

The expression of NF-*κ*B pathway-related proteins was further detected. After hyperoxia exposure for 14 days, the relative expression levels of p-NF-*κ*B p65 and p-I*κ*B*α* in the lung tissue of neonatal mice were significantly increased (*P* < 0.01) (Figures 5(a) and 5(b)). When treated with AS, the relative expression of p-NF-*κ*B p65 and p-I*κ*B*α* were obviously decreased (*P* < 0.05) (Figures 5(a) and 5(b)), indicating that artesunate may reduce the hyperoxia-induced lung injury by inhibiting the activation of the NF-*κ*B pathway. There was no significant difference in caspase-3 protein levels among the groups (Figure 5(c)).

## 4. Discussion

While advances in neonatal intensive care have improved the survival of premature infants, it is still difficult to reduce the incidence of BPD. BPD and preterm birth have been shown to have long-term effects on the lung function of children, and may even contribute to the development of pulmonary diseases [23]. In addition, the continuous effects of the current therapeutic options for BPD are unclear yet, and drugs for BPD treatment remain to be investigated [24]. Artesunate exhibited excellent anti-inflammatory and antioxidant effects in lung injury caused by other inducements [25, 26]. In the present study, we established a model of BPD neonatal mice and found that artesunate can attenuate hyperoxia-induced lung injury, after which we further investigated the potential mechanism of the therapeutic effect of artesunate on hyperoxia-induced lung injury.

MDA could be used to evaluate the level of oxidative stress as the product of lipid peroxidation, while the antioxidant enzymes SOD and antioxidant GSH played important roles in oxidative stress that can scavenge superoxide anion free radicals and protect cells from hyperoxia-induced injury [3]. Lower antioxidant levels and higher concentrations of MDA have been detected in premature infants who subsequently developed BPD [27]. Besides, alveolar simplification occurs in newborn mice exposed to hyperoxia and significantly inhibits alveolar gas transport, which is one of the pathological factors of BPD [28]. In this research, not only the increase of MDA and the decrement of SOD and GSH, the destruction of lung structure and the infiltration of inflammatory cells were also observed in lung tissues of neonatal mice after hyperoxia exposure, indicating that the hyperoxia-induced lung injury models were established successfully. After artesunate treatment, oxidative stress and hyperoxia-induced lung injury were improved in the BPD mice. On one hand, artesunate showed antioxidant properties by increasing GSH and SOD levels in the treatment of gastric mucosal injury [29], consistent with the results of our study that artesunate restored the oxidant activity and reduced the degree of lipid peroxidation induced by hyperoxia, suggesting that artesunate alleviated hyperoxia-induced lung oxidative stress in neonatal mice. On the other hand, previous studies have demonstrated that the artesunate attenuates LPS-induced lung histopathological damage [18]. H&E staining illustrated that the artesunate treatment alleviated hyperoxia-induced lung morphological changes and reduced the number of inflammatory cells as well.

Pulmonary inflammation aggravates the development of BPD, and inflammatory cytokines are related to the adverse outcome of BPD [7]. The results of ELISA demonstrated that artesunate decreased the levels of TNF-*α*, IL-6, and IL-1*β* induced by hyperoxia in the serum and lung tissue of mice, confirming that artesunate could effectively alleviate the inflammatory response caused by hyperoxia injury. Leroy et al. [30] pointed out that systemic inflammation precedes clinical symptoms in infants with BPD. The inhibitory effect of artesunate on levels of inflammatory factors induced by hyperoxia in serum suggested that it may be used for early intervention of BPD. In addition, we observed that hyperoxia-induced elevated expression of IL-1*β* in the lung tissue could be reversed by AS. The increase in IL-1*β* not only disrupts the alveolar septum and leads to abnormal pulmonary protein deposition but also inhibits the production of pulmonary vascular endothelial growth factor in neonatal mice and interferes with capillary development, which is considered to be closely related to the development of BPD in premature infants [31]. The conversion of the cytokine precursor pro-IL-1*β* to bioactive IL-1*β* is activated by the NLRP3 inflammasome [32], while the NLRP3 inflammasome is confirmed to be critically involved in the development of BPD [5]. The results of the RT-qPCR and western blot assay demonstrated that artesunate inhibited hyperoxia-induced activation and expression of NLRP3, ASC, and caspase-1, which was consistent with the findings that artesunate inhibits the NLRP3 inflammasome in other diseases [16, 33, 34]. Moreover, the NF-*κ*B pathway is involved in extensive inflammatory activation [35]. Previous studies have shown that artesunate can reduce LPS-induced acute lung injury by inhibiting the NF-*κ*B pathway [18]. In the present study, we found that hyperoxia exposure promoted the activation of the NF-*κ*B pathway, which may be associated with hyperoxia-induced lung injury and inflammation. Artesunate reduced hyperoxia-induced phosphorylation of NF-*κ*B p65 and I*κ*B*α*, suggesting that artesunate may alleviate lung injury in mice by inhibiting the activity of this pathway. There are two NF-*κ*B binding sites in the NLRP3 promoter. After LPS treatment, the NF-*κ*B subunit RelA/p65 binds to the NLRP3 promoter [36]. Thus, we speculated that the effect of artesunate on the NLRP3 may be related to the effect of artesunate on the NF-*κ*B pathway in the treatment of hyperoxia-induced lung injury, but further research is still needed.

There may be some possible limitations to this study. First, this study focused on the short-term effects of the artesunate treatment and lacked data on the long-term effects (e.g., survival). Second, this study initially investigated the effect of artesunate on hyperoxia-induced lung injury in neonatal mice via NLRP3, but did not set up a corresponding NLRP3 inhibitor group for comparison, which could be refined in the next work.

## 5. Conclusion

Our findings of the present study suggested that artesunate decreased the levels of inflammatory cytokines in the serum and lung tissues of BPD mice and alleviated hyperoxia-induced lung injury. The mechanism may be related to the inhibition of NF-*κ*B pathway activity, and suppression of the NLRP3 inflammasome, thereby restraining the expression of IL-1*β*. Our study confirms the therapeutic potential of artesunate for BPD and provides a new direction for the treatment strategy of BPD.

## Data Availability

The datasets used and/or analyzed during the current study are available from the corresponding author upon reasonable request.
